# Supplementary material for: Are carbon emissions trading and green financial instruments synergistic? -Comprehensive quantitative research based on content analysis
Source: PLoS One. 2024 Mar 7;19(3):e0298601. doi: 10.1371/journal.pone.0298601 (PMC10919601; doi:10.1371/journal.pone.0298601)
Supplement: S1 Data — (ZIP) [file pone.0298601.s001.zip › Data/地级市Z_F工作报告.zip!/╕╩╦α/╒┼╥┤╩╨/└┤╫╘╣1⁄2╓┌║┼í╛┬φ┐╦╩2╛▌═°í┐_2014.docx]

各位代表：

　　现在，我代表市人民政府向大会报告工作，请予审议，并请各位政协委员和列席人员提出意见。

　　2013年工作回顾

　　2013年，在市委的坚强领导下，全市上下以生态文明引领经济社会发展全局，牢牢把握率先实现转型跨越的总基调和做大做靓宜居宜游的战略基点，着力培育壮大宜居宜游首位产业，统筹推进民生保障和社会治理，基本完成了市三届人大二次会议确定的目标任务。实现生产总值336.9亿元，增长11.8%。完成固定资产投资275.2亿元，增长31.3%。实现社会消费品零售总额106亿元，增长14.5%。大口径财政收入和公共财政预算收入完成40.1亿元、16.7亿元，分别增长32.93%和32.06%。城镇居民家庭人均可支配收入、农民人均纯收入增长10.3%和12.8%，分别达到15877元和8465元。

　　──发展速度明显加快。省政府考核的主要经济指标绝大部分高于全省平均水平，8项增速位次前移。其中生产总值、公共财政预算收入、固定资产投资等核心指标增速分别位列全省第9、第3和第3位，较上年分别前移5位、5位和4位。

　　──产业结构持续优化。三次产业占GDP的比重调整为27.6:35.7:36.7，三产固定资产投资占比和对财政税收的贡献率均超过50%。旅游综合收入、接待国内外游客人数、文化产业增加值增速位居全省前列，均创历史最高水平。宜居宜游首位产业的地位更加强固，一产稳固、二产创新、三产扩容的“三二一”内生增长型格局初步形成。

　　──开放开发强力推进。招商引资签约资金超过1000亿元，项目履约率大幅提升，到位资金实现翻番。11家国内外知名大企业、大集团入驻张掖发展，一大批招商引资项目顺利实施，开放开发水平明显提高。

　　──社会大局和谐稳定。城乡居民收入快速增长，生活水平有了新的提高。各级财政用于改善民生的投入达77.6亿元，一大批民生项目相继建成并发挥作用，社会保障能力进一步提升。安全生产形势持续好转，“四项控制”指标全面下降，群众合理诉求有效解决，呈现出矛盾减少、大局稳定、社会和谐的良好局面。

　　一年来，我们主要抓了以下工作：

　　(一)全力抓好项目建设，"3341"工程取得新成效。开工建设各类项目668项，完成投资392.3亿元，其中，亿元以上项目175项，政府投资项目258项，到位中央及省级预算内资金8.1亿元。浙江正泰100兆瓦及黑河水电100兆瓦光伏发电一期、山泥集团2500吨熟料生产线、临泽宏鑫30万吨铁锰精选、城市天然气利用等340个项目建成投入运行。安达公司60万吨煤化工、祁连山集团200万吨干法水泥等80个工业项目前期工作取得实质性进展。

　　(二)加快转变发展方式，产业培育实现新突破。坚持把培育壮大宜居宜游首位产业与大力发展现代农业、生态工业紧密结合，优势主导产业发展步伐明显加快。一是宜居宜游首位产业不断壮大。大景区建设取得显著成效，丹霞国家地质公园、国家湿地公园等70个新建、续建重点旅游项目加快推进，完成投资32.2亿元。国家4A级旅游景区新增4家，达到9家。中国·张掖汽车拉力锦标赛、全国露营大会、环青海湖自行车赛、“湿地之夏”金张掖旅游文化艺术节、多彩张掖户外运动之旅等重大赛事活动成功举办。央视等主流媒体相继聚焦报道张掖。文化产业发展提速，山丹马场国际文化旅游度假区等30个文化产业项目开工建设，完成投资32.5亿元，开发祁连玉等文化产品45类，文化企业发展到749家。宜居宜游配套产业快速发展，新张掖国际商贸城、临泽浙商文化商业广场、高台汉唐文化商业街、民乐商业步行街等商流项目加快建设，完成投资7.9亿元。交通运输、餐饮住宿、信息咨询、健康养老等服务业发展迅速，完成第三产业增加值123.5亿元，增长12.5%。二是现代农业稳步发展。完成农作物播种面积407.4万亩，粮食生产再获丰收，总产达128万吨。大力发展节水、高效、绿色现代农业，高原夏菜、设施农业、马铃薯等特色产业规模效益同步提升。张掖市和甘州、临泽、高台3县(区)获得国家级玉米制种基地授牌。草畜产业快速发展，牛羊饲养量分别达到88.8万头和444.1万只。制定出台建设农产品安全大市《实施意见》，标准化、无公害生产面积达到302万亩和230万亩，“张掖肉牛”地理标志证明商标评定公示。开工建设沅博肉羊肉牛、爱福食用菌、三维豆制品等12个投资上千万元的农业产业化重点项目，完成投资18.3亿元，新增农产品加工能力3.8万吨，加工转化率达到57%。甘州区进入全国农业改革与建设试点示范区行列，民乐被确定为全省唯一国家农业综合开发现代农业园区试点项目县。绿洲现代农业试验示范区开工建设项目67项，完成投资4.6亿元。农业社会化服务体系不断完善，产业化经营组织达526家。流转土地91.1万亩，耕播收综合机械化水平提高到71.3%。农村集体土地所有权发证基本完成。输转劳动力28.9万人，实现劳务收入39.6亿元。三是生态工业加快发展。制定出台加快生态工业发展《实施意见》，“两个园区、千亿产值”的奋斗目标进一步明确。开工建设重点工业项目129项，建成投产72项，全部工业完成投资107亿元。20户矿山企业恢复生产。新增规上企业41户、销售收入上亿元的企业5户。完成工业增加值88.5亿元，增长15.8%;实现规上工业增加值70.5亿元，增长16.1%。张掖工业园区晋升为国家级经济技术开发区，民乐生态工业园区规划修编全面完成。两个园区新增企业47户，完成投资23.9亿元。大力发展循环经济，3个工业园区和昆仑公司等5户企业被确定为省级循环经济示范园区和示范企业。关停产能过剩、安全隐患突出的小企业11户，淘汰落后产能52万吨，万元生产总值能耗下降4.25%。

　　(三)加大开放开发力度，创新发展增添新活力。完善招商引资优惠政策和奖励办法，制定出台项目全程代办暂行规定，推行并联审批制度，建立项目审批快速通道，全民招商氛围更加浓厚，合力进一步增强。精心筛选10大类700多个项目开展定向精准招商，全年签约招商项目205项，总投资1141.2亿元，到位资金241.2亿元，增长102.11%。甘州东银3万头肉牛养殖、临泽长业30万吨生物有机肥料加工、高台环保桥20兆瓦光伏发电、山丹闽龙食用菌基地建设、民乐城市综合体开发、肃南中能100兆瓦光伏发电等重点招商项目落地开工。积极与万达、横店、愿景等一批国内知名企业和美国杜邦、泰国正大、瑞典沃尔沃等世界500强企业对接洽谈，达成合作意向。与内蒙古阿拉善盟签订区域合作协议。非公经济快速发展，增加值占GDP比重达到54.2%，提高2.2个百分点，市场主体增加到5.2万户。上源农业、鸿泰种业在上海股权托管交易中心挂牌上市。

　　(四)强化生态保护建设，可持续发展能力不断增强。实施张掖国家湿地公园保护与恢复等各类生态项目32项，完成投资8亿元。《祁连山生态保护与建设综合治理规划》、《黑河湿地国家级自然保护区总体规划》获批，《黑河流域综合治理规划》、《张掖国家地质公园规划》通过评审。张掖被确定为全国资源再生型城市和水生态文明建设试点市，临泽、民乐被列为国家沙化土地封禁保护试点县。黑河湿地湖泊纳入全国重点治理规划。西北地区人工增雨雪作业基地建设、祁连山生态功能区张掖生态保护与综合治理等项目前期工作进展顺利。湿地博物馆、祁连山滑雪场、湿地公园观光航道建成开放，飞禽保护区基本建成。重点公益林生态效益补偿工作扎实开展，天然林保护、三北防护林、退耕还林等生态建设工程深入实施，完成人工造林8.2万亩、封育7.8万亩。

　　(五)加强基础设施建设，发展条件进一步改善。开工建设城市基础设施项目214项，完成投资113.9亿元。张掖宾馆、甘州府城等重点项目和市区热电联产集中供热、三水厂、污水处理二期等98项市政公用项目顺利实施。加快推进5县城建设，全市城区面积扩大到148.8平方公里，城镇化率提高到38.7%。投资7.3亿元，完成河西走廊国家级高效节水灌溉示范、大中型灌区节水改造、病险水库除险加固、中央财政小型农田水利、中小河流治理等各类水利项目建设任务。整理土地8.9万亩，新增耕地2万亩。交通运输业完成投资10.5亿元，张扁高速等3条233公里道路建设纳入国网规划，黑河大桥、东六公路完成前期工作，甘祁公路一期和丹霞景区景观大道、快速通道完成路基工程，建成农村公路645公里。兰新铁路第二双线完成投资31.5亿元，张掖、临泽、高台、民乐4个高铁车站主体工程基本建成。张掖-西安-广州航班开通，机场航线不断优化，全年运送旅客4.3万人次。电力、通讯、天然气等基础设施建设进程加快，高台330千伏变电站投入使用，西气东输三线工程张掖段完成建设任务。

　　(六)统筹推进社会事业，和谐发展开创新局面。实施社会事业项目126项，完成投资14.3亿元。省上确定的9项23件实事和市上确定的11件实事基本落实。加强科技创新、成果转化和服务能力体系建设，张掖市和甘州、民乐分别荣获全国科技进步先进市、县(区)称号。投资3亿元改善办学条件，学前教育、义务教育、高中阶段教育和高等教育协调发展。投资2.4亿元加强医疗卫生设施建设，群众就医条件进一步改善，医疗保健水平不断提高。人口自然增长率控制在5.24‰，出生人口性别比与第六次人口普查相比，下降19.96个点。国家公共文化服务体系示范市建设启动实施。市级“三馆”主体完工，张掖大剧院开工建设。山西会馆、总兵府、钟鼓楼、明粮仓、福音堂医院旧址等重点文物修缮工程扎实推进。深入实施全民健身计划，群众性体育活动广泛开展。落实创业带动就业各项优惠政策措施，发放小额担保贷款3.9亿元。城镇新增就业3.8万人，登记失业率控制在2.5%以内。提高最低工资、城乡低保、五保供养、养老金补助标准及住房公积金缴存比例，保障性住房和农村危房改造任务全面完成。《张掖市祁连山浅山区区域发展与扶贫攻坚规划》通过省上评审，甘州、肃南、山丹、民乐、高台5县(区)纳入“插花型”贫困片带，山丹、肃南被确定为全省扶贫开发工作试点示范县和交通先行示范县。双拥共建、外事侨务、机构编制、扶残助残、审计统计、工商物价、环保质监、地震气象、老龄妇幼、史志档案等工作都取得了新的明显进步。

　　(七)加强和创新社会治理，科学发展迈出新步伐。严格落实“八项规定”，深入推进机关“效能风暴”行动，取消、下放和调整行政审批事项62项。健全完善电子监察系统，市县区政务中心便民服务内容更加充实、效率不断提高。成立市公共资源交易中心，积极推进公共资源配置市场化改革。规范党政机关、事业单位办公用房管理，调整腾退6.1万平方米。市本级及各县(区)财政预决算通过网络全部向社会公开，“三公”经费及部门决算公开试点工作有序推进，市本级压缩“三公”经费11%。合理确定市、区两级事权，城市规划建设管理体制进一步理顺。严格执行政府议事规则和工作程序，自觉接受人大法律监督、政协民主监督、专门机关监督、新闻舆论监督和社会各界监督，112件人大代表建议、188件政协提案全部办理答复。健全完善应急制度体系，应对自然灾害及突发公共事件能力进一步增强。强化安全生产监管，加大隐患排查整治、打非治违、人员培训力度，扎实开展农村火灾隐患集中整治活动，事故多发势头得到有效遏制。深入推进“平安张掖”、“法治张掖”建设，严厉打击各类刑事犯罪，下大力气解决信访矛盾纠纷，强化食品药品安全监管，严格整治规范市场秩序，依法管理民族宗教事务，有力地维护了社会大局和谐稳定。

　　各位代表，在国内外形势错综复杂、经济下行压力加大的情况下，全市经济社会发展取得这样的成绩实属不易。这是省委、省政府亲切关怀、坚强领导的结果;是市委运筹帷幄、科学决策的结果;是市人大、市政协和社会各界有效监督、大力支持的结果;是全市各族干部群众开拓创新、团结奋斗的结果。在此，我代表市人民政府，向关心支持张掖发展的社会各界人士，向奋战在全市各条战线上的人大代表、政协委员、干部群众，向各民主党派、人民团体、中央和省属驻张单位，向离退休老同志、驻张部队、武警官兵、公安干警表示衷心的感谢并致以崇高的敬意!

　　我们也更为清醒地看到，经济社会发展和具体工作中还存在不少困难和问题。主要表现在：大项目、好项目储备不足，新开工项目少，产业性重大项目接续不足，经济增长的内生动力不强;城市居民收入水平偏低，农民持续快速增收难度加大，扩大内需和消费动力不足;企业产成品库存增加、流动资金趋紧、经营效益下滑，工业经济“短腿”的局面不仅没有从根本上改善，而且还有继续下滑的可能;财政收入结构不合理，一次性收入和非税收入占比过高，税收增长乏力，刚性支出明显增加，收支矛盾更加突出，保障民生的能力依然较弱;政府职能转变不到位，审批事项多、程序繁杂，办事效率不高，一些部门巧立名目乱收费、乱罚款的问题依然存在，直接影响投资环境优化和市场主体发育成长;部分干部思想观念、政策水平、工作作风、执行能力不能适应深化改革、转型发展的形势要求;形式主义、官僚主义、享乐主义和奢靡之风不同程度存在，在一些领域还比较突出。对此，我们将采取有力措施，切实加以解决。

　　2014年工作任务

　　2014年，政府工作的总体要求是：坚持科学发展主题，以党的十八届三中全会精神为指导，紧紧围绕“建设丝路明珠金张掖、实现幸福美好家园梦”的奋斗目标，秉持“立于生态、兴于经济、成于家园”的理念，坚持把发展生态经济作为转变发展方式的基本途径，把建设生态经济功能区作为经济结构战略性调整的主攻方向，把培育壮大宜居宜游首位产业作为拉动经济增长的强力引擎，持续强化基础设施建设、生态建设、文化建设和产业培育，深入推进“大景区、大互市、大博物馆、大运动场”建设，更加注重深化改革、开放开发、民生改善和安全安定，努力开创经济社会转型跨越发展的新局面。

　　经济社会发展的主要预期目标是：生产总值增长11.5%以上。固定资产投资增长26%以上。社会消费品零售总额增长14%以上。公共财政预算收入增长14%以上。城镇居民人均可支配收入和农民人均纯收入均增长13%以上。居民消费价格涨幅控制在3.5%以内。城镇化率提高1.6个百分点以上。全面完成省政府下达的各项目标任务。

　　重点抓好以下七个方面的工作：

　　(一)强化宜居宜游首位产业主导地位，增强经济发展内生动力

　　以建设国际旅游度假名城和区域游客集散中心为目标，加快培育壮大宜居宜游首位产业，着力打造中国地貌景观大观园、暑天休闲度假城、丝绸之路古城邦、户外运动体验区的靓丽名片，推动旅游文化龙头产业发展提速、比重提高、水平提升。

　　1.加快大景区建设。修编完成张掖国际旅游度假名城建设暨旅游业发展规划，加快编制大景区发展总体规划、功能片区规划和重点景区建设性详规。丹霞国家地质公园开园揭牌，丹霞七彩镇、硖口长城文化游览区、马文化产业园、山丹马场鸾鸟湖草上乐园等33项重点旅游项目开工建设;抓好国家地质公园快速通道、景观大道，大沙河、大湖湾、扁都口、焉支山、裕固风情走廊等34项旅游续建项目;加快通往4A级景区二级公路、重点景区道路、旅游专线通道和山丹至中牧马场公路改建等旅游交通项目建设，完善游客服务中心、景区公厕等配套服务设施。新创建4A级旅游景区3家，新增年接待游客达到百万人次以上的大景区2家以上。启动丹霞地质公园、马蹄寺、焉支山等重点景区创建5A级景区资源评估工作。

　　2.延长旅游产业链条。启动建设展示丝路文化的“大博物馆”，组织实施丝绸之路古城邦、骆驼城古遗址博物馆等重点文化项目。加快建设具有国际知名度的“大运动场”，开工建设以体育公园、公共足球场为重点的体育场馆，加快推进滨河新区自驾游营地、国际乡村俱乐部、国际赛车城、西游文化主题公园、露营基地、国际滑雪场等经营项目建设，办好中国·张掖汽车拉力锦标赛、冬季山地户外挑战赛、户外运动嘉年华等重大赛事。积极开发演艺娱乐、滑雪滑冰、森林旅游、湿地养生、黑河漂流、登山健身、赛马赛车等休闲度假产品，完善宾馆饭店、购物中心、租车公司等服务设施，提升旅游产业综合效益。加强宣传推介，组织开展“张掖旅游全民宣传行动”，营造人人当代言人、个个做宣传员的强大声势和良好氛围。办好第四届敦煌行·丝绸之路国际旅游节开幕式。全年接待游客1000万人次以上，实现旅游综合收入50亿元以上，占GDP的比重达到13%以上。

　　3.提升服务管理水平。加快景区服务设施、旅游咨询和信息服务体系建设，启动“智慧旅游”建设工作。深入开展旅游服务管理能力提升行动，认真执行《旅游法》和各项管理服务标准，出台规范市场秩序的地方标准，及时受理旅游投诉，严厉查处、依法打击欺客宰客、欺行霸市等有损张掖旅游形象的人和事，防止“一个老鼠坏一锅汤”。新创建四星级酒店3家以上，星级酒店达到36家以上。加大人才培养力度，引进旅游专业人才100名以上，培训旅游从业人员4000人次以上，持证上岗率达到70%以上。

　　4.推动旅游文化深度融合。深入挖掘张掖历史文化特质，强化文化产业发展的载体和平台，为旅游强魂增色。注重对重大旅游项目的文化包装，培育提升核心竞争力。加大祁连玉、剪纸、根雕、刺绣等旅游文化商品开发力度，积极培育文化产业市场主体，新发展文化企业120家，文化产业增加值占GDP比重提高到1.85%以上。积极开展华夏文明传承创新区建设，加强文物遗址、传统建筑、人文景观、民族民俗的保护利用，健全完善非物质文化遗产陈列展示、传承保护和创新发展体系。扎实推进国家公共文化服务体系示范市创建工作，落实公共文化服务场所免费开放政策。加快数字文化馆、图书馆、博物馆、科技馆建设，建成张掖丹霞地质博物馆、现代农业展览馆。以“乡村舞台”建设为重点，吸引社会资金加大投入，建设综合性文化服务中心。加快实施市广播电视台高清数字化和全市有线电视双向化改造工程。

　　5.培育发展新型物流产业。抢抓国家建设丝绸之路经济带历史机遇，发挥独特而重要的区位交通优势，大力发展新型物流产业，加快建设商贸流通“大互市”。编制完成物流业发展规划，力争更多项目进入国家和省级物流规划。积极推进张掖国际物流园区、绿洲现代物流园区、火车站物流园区等重点项目建设，启动建设山丹、民乐超大规模高速服务区和物流园。加快新张掖国际商贸城、天佑金三角商业广场等商流基础设施建设。采取政府扶持、市场运作的方式，举办丝绸之路经济带特色商品及绿色农产品张掖交易会，不断扩大品牌效应。深化与阿拉善盟等周边地区合作，以良好的生态、便捷的交通、优质的服务吸引各类生产要素向张掖聚集。大力发展综合市场、连锁经营、电子商务、信用服务等新型业态，鼓励发展第三方物流配送。健全完善城乡商业网络体系，深入实施“万村千乡市场工程”、“农超对接”项目，加快批发零售、再生资源回收等专业市场建设。

　　(二)加快建设农产品安全大市，提升现代农业发展水平

　　严格落实建设农产品安全大市《管理办法》，充分发挥比较优势，集中力量推动绿色有机现代农业加快发展，促进农业增效、农民增收、农村繁荣。

　　1.壮大优势主导产业。加大农业结构调整力度，大力发展玉米制种、畜禽养殖、高原夏菜三大优势主导产业，积极培育中药材、马铃薯、食用菌、林果四大区域特色产业，扩大生产规模，提升比较效益。发展玉米制种70万亩、高原夏菜50万亩、中药材40万亩、马铃薯45万亩、食用菌3000万袋以上。新建规模化养殖小区60个，肉牛、肉羊饲养量分别达到100万头和500万只。争取张掖国家级玉米制种基地建设规划通过审批，启动实施国家级玉米制种基地甘肃30万亩建设项目，确保国家级杂交玉米种子生产基地病虫害预警与控制中心建设、国家农作物制种大市大县财政转移支付专项奖补政策争取工作取得实质性进展。以现代农业科技试验示范为导向，修编完善《张掖绿洲现代农业试验示范区建设规划》，强化政策引导，吸引高等院校、科研院所、龙头企业开展高值农业试验示范推广，开工建设中国农大张掖现代农业科研教学实验站、甘肃农大科技服务专家院，加快制种、草畜、果蔬等农产品加工园区建设。

　　2.加快农业产业化进程。围绕特色优势农产品精深加工，大力招引和扶持龙头企业，培育一批市场竞争力强、社会知名度高的国家级农业产业化龙头企业。建成沅博肉牛肉羊屠宰、亚兰中草药饲料化利用等投资上千万元的农业产业化重点项目12项，农产品加工转化率达到58%。立足原料优势，加快发展饲料、有机肥料等加工业，打造从种植、养殖、加工到辅料处理的完整产业链。完善农产品流通体系，加快发展冷链物流和生鲜农产品配送，建设改造一批农产品批发市场和物流产业园。建立农产品网上交易平台，设立一线城市展销档口，开拓沿海和口岸市场，鼓励企业参加全国及国际性展览展销会。培育农村土地承包经营权流转市场，推进土地规范有序流转。深化国有农林牧场改革，支持农民以承包经营权入股参与农业产业化经营，激活生产要素，提高土地经营效益。大力发展合作经济组织，积极培育专业大户、职业经理人、职业农民、家庭农场，开展集约化、专业化、组织化生产，构建新型农业产业化经营体系。

　　3.打造绿色有机农产品品牌。坚持绿色发展取向，加强宣传引导，把发展绿色有机现代农业转化为全社会的自觉行动。全面落实“一严格、五禁止”措施，按照绿色有机标准统筹规划全市农产品生产基地。推行农产品产地准出和市场准入制度，健全完善农产品质量追溯和检测检验体系，稳步扩大有机农产品生产面积，标准化、无公害农产品生产面积分别稳定在300万亩和230万亩以上。积极开展无公害、绿色、有机农产品产地认定、产品认证和地理标志登记，新认证绿色有机食品10个以上。选择一批重点产业、重点企业、重点产品开展品牌创建，实施原产地保护和商标注册，努力打造一批知名度高、带动力强、辐射面广的绿色有机农产品品牌，不断提升市场竞争力。

　　4.加强农田水利基础设施建设。把农田水利建设作为改善农业生产条件的重中之重，加大项目争取和投入力度，加快重点水源配置工程前期工作进度，争取实施临泽红山湾水库及山丹东沟、西沟、大口子河水库等重点水源工程。继续抓好河西走廊国家级高效节水、中央财政小型农田水利和甘州区规模化节水增效示范等项目，开工建设临泽、高台、山丹3县以色列政府贷款高效节水项目，新发展高效节水面积16万亩。深入实施灌区节水改造、病险水库除险加固、中小河流治理、重点河段整治等项目，切实提高防洪减灾能力。积极做好农村水电、水资源费及抗旱防汛经费补助等项目的争取工作。加快农村饮水安全项目建设，年内解决20万人饮水安全问题。

　　(三)加快发展生态工业，夯实财源和经济增长基础

　　坚持生态工业的发展方向，创新发展模式，加大政策扶持、金融支持和招商引资力度，加快工业园区和重大项目建设，扩大工业投资规模，提升工业经济效益。

　　1.加大生态工业发展力度。把工业经济作为壮大财源的基础，作为吸纳就业、聚集要素的平台，作为宜居宜游首位产业的重要支撑，坚定发展信心，加快发展步伐。在价值取向上，坚持生态立市，把资源生态化、生态产业化、产业集群化作为发展生态工业的主攻方向，以最小的环境代价和最合理的资源消耗，获取最大的经济社会效益。在发展路径上，突出企业集约发展、资源循环利用，一方面，把乱批园区、乱建企业坚决管住、管好;另一方面，把张掖经济技术开发区和民乐生态工业园区做大做优做强，实现不破坏自然生态、不污染人居环境的生态工业可持续发展目标。在工作举措上，充分发挥政府在政策支持、环境营造、平台打造方面的职能作用，坚持资源导向与市场导向并举，加大招商引资力度，激发各类市场主体投资生态工业的积极性，形成发展生态工业的强大合力。

　　2.提升园区建设水平。按照“两个园区，千亿产值”的目标要求，全面推进张掖经济技术开发区和民乐生态工业园区建设。修编完善园区规划，加快道路、电力、供水、排污等基础设施建设，改善企业入驻条件。市上安排2000万元，甘州区、民乐县分别安排1000万元专项资金扶持园区建设。落实支持园区加快发展的政策措施，建立引导优势资源、优势产业、优势项目向“两个园区”集中的利益共享机制。搭建公共融资和项目信息共享平台，加快形成基础设施配套、生产性服务业健全的园区保障体系。

　　3.培育壮大优势产业。依托资源禀赋，紧盯市场需求，完善产业体系，延伸产业链条，努力做大做强新能源、新材料和特色农产品深加工为主的主导产业。以风电、光电、生物质能和火力发电为重点，着力推动高台高崖子滩、甘州平山湖等5个“百万千瓦级”风光电基地建设，新开工18万千瓦光电、15万千瓦风电等新能源项目，全年装机达到80万千瓦。以冶金新材料、新型建材、化工新材料为重点，全力抓好年处理100万吨钨尾矿回收利用、20万吨锰硅合金等新材料重大项目建设，完成投资25亿元。以玉米种子、畜产品、奶制品加工为重点，建成秋乐3万吨种子加工、金昱50万只肉羊屠宰、金明生物新型食品加工等29项特色农产品深加工项目，完成投资12.3亿元。立足生态、交通、土地、劳动力等方面的比较优势，全力招引制造业、高科技企业入驻张掖，不断提高工业经济整体实力。

　　4.大力发展循环经济。积极争取张掖经济技术开发区循环化改造示范试点项目通过国家评审。加快节水节能技术改造，组织年耗电1000万千瓦时以上的企业实施电机能效提升工程。扎实推进循环经济试点示范工作，争取2户企业纳入省级循环经济试点范围。积极做好国家及省级淘汰落后产能项目申报工作。强力推进煤炭资源整合重组，依法关停能耗高、污染重、安全隐患突出的小企业。

　　(四)加快新型城镇化建设步伐，统筹城乡一体化发展

　　牢牢把握新型城镇化的特点和规律，坚持以生态文明引领城市建设，突出产业聚集、功能完善、特色彰显和经营管理，形成宜居宜游、宜商宜业、城乡互补、协调发展的城镇化建设新格局。

　　1.强化规划引导作用。健全完善规划编制审查、公示、听证和监督制度，切实维护规划的权威性和严肃性。按照两个“1+5”生态城市框架和“两城两心”的城市定位，分解落实《张掖市宜居宜游城市发展规划纲要》任务，编制完成张掖市城镇风貌规划和历史文化名城保护重点片区规划，修编老城区、滨河新区、西南片区、工业园区控制性详细规划，推广应用城市三维数字影像系统，建立老城区地下管线数据采集、数据库及管线管理信息系统。加快推进5县城总规、详规、控规和城乡交通发展规划修编，完成所有建制乡镇、公路沿线及大景区周边乡村规划，用高水平规划指导新型城镇化建设。

　　2.完善城市基础设施。开工建设总投资39.8亿元的城镇化项目93项。改拓建城市道路34条32.1公里、供水管道98.3公里。实施临泽、高台、民乐3县城市垃圾收集中转工程，完成5县城区集中供热工程，建成市区热电联产项目。加快张掖大剧院、高铁客运站广场、公交客运枢纽等项目建设进度，增强城市综合服务功能。

　　3.提升城市经营管理水平。适应新型城镇化加快发展、城市资源聚集作用日益显现的形势要求，树立经营城市理念，全方位整合、聚集、利用城市资源，形成推动经济发展的强大动力。进一步完善“市上主导规划、参与建设，区上主导建设管理、参与规划”的城市管理新机制，集中力量开展违章建筑拆迁攻坚和以公共卫生为主的环境整治、以退市还路为主的市场整治、以美化亮化为主的风貌整治行动，建设重点精品街区。加强城市交通综合管理，实行公共场所停车泊位市场化运作，严肃整治乱停乱放等违章违规行为。严格落实环卫保洁人员定时清扫、单位住户“门前三包”、社会公益性岗位人员全天候维护的城市环境卫生管理制度。加强物业管理，切实解决居民反映的突出问题。广泛开展文明城市、文明社区、文明市民评选活动，创建洁净、舒适、优美、文明的生活环境。在全市范围内开展以张掖城市精神大讨论、城市风貌大整治、行为陋习大曝光、市民素质大提升为主要内容的“城市形象提升行动”，增强市民的自豪感和城市管理的内在动力。

　　4.加强历史文化名城保护。按照“历史为根、文化为魂、西部风貌、张掖特色”的定位，启动华夏文明传承创新区和历史文化名城名镇名村保护利用板块试点建设工程。全面梳理、分解落实历史文化名城保护建设任务，老城区以“降低密度高度、提升文化品位、彰显城市特色”为导向，实施渐进式、渗透式改造建设，启动实施文庙巷、西来寺巷、明粮仓等重点片区改造和钟鼓楼、西夏国寺、总兵府等重点文物周边建筑风貌改造工程。经过5年左右的持续建设，塑造“历史文化名城”和“丝绸之路重镇”的城市形象。

　　5.加快小城镇和新农村建设。坚持规划先行，坚持“四化”方向，统筹城镇化与新农村建设，加快城乡一体化进程。启动重点乡镇道路、供排水、供热等基础设施建设，不断提升小城镇和示范村辐射带动能力。完善已建“四化”新农村设施功能，坚持不懈地整治村容村貌，引导农民养成健康文明的生活方式，创建一批“美丽乡村”。以国、省主干道沿线和大景区周边为重点，启动新一轮50个新农村“四化”示范村建设工程，改造农村危房1.5万户。

　　(五)持续强化项目建设，提高经济发展质量效益

　　紧盯国家产业政策和投资趋向，加大项目、资金争取力度，加快建设一批事关长远发展的重大项目，推动全社会固定资产投资持续快速增长。

　　1.强力推进"3341"项目工程建设。紧抓甘肃建设国家生态安全屏障综合试验区重大机遇，用好华夏文明传承创新区和国家公共文化服务体系示范区创建平台，围绕国家产业政策、区域经济政策，谋划、论证、储备、争取一批事关可持续发展的大项目、好项目。紧紧围绕丝绸之路经济带建设，谋划争取节点城市建设、大型物流园区、会展经济等项目;围绕国家产业布局调整和投资趋向，重点谋划争取特色农产品加工、矿产品加工、煤化工等项目;围绕国家生态安全屏障试验区建设，谋划争取一批重大生态保护和建设项目，力争国家投资额度持续增长。全力推进祁连山生态保护与建设治理工程、花草滩循环经济产业区、安达能源16万吨化工新材料等522项在建项目，争取更多项目建成投入使用。健全完善服务重点项目、重点企业“绿色通道”，全面落实重大项目建设“五个一”责任制和“代办制”，进一步提升项目服务水平，提高项目落地率和开工率。

　　2.加大招商引资力度。严格落实招商引资工作责任制。依托工业园区、优质资源和重大项目，积极开展定向招商、精准招商和以商招商，切实提高项目成功率和资金到位率。瞄准世界500强、国内500强、行业百强和大型央企，全方位开展项目推介，加强交流合作，承接产业转移，加快“引强入张”步伐，力争全年招商引资签约资金1200亿元以上，到位资金超过300亿元。

　　3.加快交通电力设施建设。以建设东西畅通、南北贯通，铁路、公路、机场联动的立体交通枢纽为目标，积极配合做好兰新铁路第二双线、兰州至张掖三四线铁路建设。支持张掖机场优化航班航线，大力发展通用航空。开工建设甘州城区经民乐六坝至山丹东乐一级公路、黑河跨河大桥和山丹长城驿服务区、张掖中心客运站及高台、山丹、丹霞景区客运站。力争张阿公路、祁连山腹地公路等7条1455公里公路纳入省网规划。建成建制村通畅工程500公里以上，村级停靠站点100个，公路通畅率提高到80%以上。加快重点项目申报，健全完善输电网络，启动建设6县(区)农网改造升级工程和民乐生态工业园区输变电线路改造等电力项目。实施信息网络宽带扩容升级工程，全面提升通讯信息和规上企业数字化建设水平。

　　4.促进金融和民营经济快速发展。加快金融创新发展，增强金融支撑能力。引导金融机构扩大信贷规模，提高存贷比，贷款余额达到360亿元以上，存贷比提高到70%以上，使更多的金融资金用于支持地方经济社会发展。引进交通银行、招商银行设立分支机构，扶持村镇银行、小额贷款公司和担保机构加快发展，为民营经济健康快速发展提供更加有力的资金支持。成立上海股权托管交易中心张掖中小企业孵化中心，鼓励企业积极发展债券融资和股权融资，提高直接融资比重，争取实现市城投发债和黑河水电上市目标。制定民营经济进入特许经营领域的具体办法，支持发展非公有资本控股的混合所有制企业，推动非公经济进入基础设施、市政公用事业、社会事业和金融服务等领域。深入推进“扶助小微企业专项行动”，着力培育扶持成长性好的小型企业壮大规模实力。健全完善中小企业公共服务平台，建成具有一定规模的创业孵化基地。

　　(六)持续扩大生态比较优势，进一步强化发展基础

　　从张掖特殊而重要的生态地位出发，引导全社会特别是各级干部牢固树立以生态文明引领科学发展的理念，建立健全保护生态、改善生态、永续发展的体制机制。整合人才、平台资源，组建中国张掖生态科学研究院，为甘肃建设国家生态安全屏障综合试验区提供科研支撑。抢抓国家加快建设生态文明和省上打造生态战略平台历史机遇，加大生态保护和基础设施建设力度，夯实可持续发展基础。积极落实资源有偿使用和生态补偿制度，加快推进祁连山、黑河生态屏障试验区建设。组织实施好黑河湿地恢复与治理二期、退耕还林、三北五期、生态公益林、草原生态保护补助政策及退牧还草等重点生态工程和沙化土地封禁保护试点项目，争取实施黑河湿地国家级自然保护区基础设施建设、张掖市森林重点火险区综合治理二期工程、黑河流域林业有害生物防控体系建设等项目。全力做好祁连山生态保护与建设、黑河流域综合治理等重大生态项目前期工作，争取早日启动实施。深入推进生态文明试点示范市建设，认真抓好工业污染、城市大气污染防治工作，加大重金属污染治理力度。加强农村环境保护，重点解决饮用水源地保护、生活垃圾和污水处理、“白色污染”、畜禽养殖污染防治等问题，不断改善人居环境。精心筹办绿洲论坛，进一步提升对外影响力。

　　(七)持续保障和改善民生，提高社会治理创新能力

　　把促进城乡居民增收放在更加重要的位置，切实解决事关人民群众切身利益的突出问题，全力办好为民承诺的实事，使发展成果更多更公平地惠及民生，维护大局稳定，促进社会和谐。

　　1.着力增加城乡居民收入。最大限度挖掘农业增收、非农产业增收、转移性和财产性增收潜力，促进农民收入稳定快速增长。加大劳务输转力度，维护农民工合法权益，完成转移就业培训3万人，输转劳动力26万人以上，实现劳务收入40亿元以上。选择1-3个基础条件好的乡村，积极稳妥地开展农民住房、土地抵押、担保、转让试点工作，探索激活农业生产投资和农民资产变资本的有效途径。把就业作为民生之本，实施更加积极有为的就业创业政策，全力帮助大中专毕业生、城乡富余劳动力等重点群体实现充分就业。建立完善企业职工工资正常增长机制，落实国家出台的各项津补贴政策，促进干部职工和城镇居民收入稳定较快增长。

　　2.加快扶贫攻坚步伐。按照省上"1236"扶贫攻坚行动的统一部署，以率先实现整体脱贫为目标，紧抓祁连山浅山区贫困片带整体纳入全省“插花型”扶贫范围的重大政策机遇，突出产业培育、基础设施建设和人口素质提高，争取组织实施交通水利、电网升级、危房改造和易地搬迁项目，深入推进“双联”行动，构建大扶贫格局。力争贫困地区农民人均纯收入增幅高于全市平均水平2个百分点，贫困人口减少20%，贫困发生率降低到5%以下。

　　3.提高社会保障水平。扩大社会保障覆盖范围，认真做好新型农村和城镇居民社会养老保险合并工作，稳步推进城镇常住人口基本公共服务全覆盖。提高城乡低保补助标准，进一步扩大大病救助范围。落实城镇低收入家庭住房保障政策，新建保障性住房11626套96万平方米，完成8067套78.5万平方米棚户区改造任务。加快全国综合养老示范基地、城乡社区日间照料中心、老年福利服务中心、农村敬老院等养老服务场所建设。推进殡葬管理改革，加快殡葬服务设施建设。加强国防教育、动员和后备力量建设，积极做好双拥共建、优抚安置和扶残助残工作，大力发展社会福利和慈善事业。健全完善防灾减灾和公共安全应急救援体系，保障人民群众生命财产安全。

　　4.统筹发展社会事业。全面落实教育优先发展战略，推进各类教育均衡发展。建立完善产学研相结合的技术创新体系，抓好张掖创新型试点城市和省级可持续发展实验区建设。高质量完成第三次全国经济普查任务。深化医疗卫生体制改革，巩固提升基层医疗卫生机构基本药物零差率销售成果，进一步完善公共卫生防控体系。落实计划生育优惠政策，综合治理出生人口性别比，推动人口和计划生育工作和谐发展。完成甘州中学、临泽职业中专实训楼等52项教育项目及妇幼保健院和卫生监督机构等38项卫生项目建设任务。加快数字化影院、标准化文化站、社区和乡村文化小广场等重点公共文化工程和文化体育项目建设，市级“三馆”建成投入使用。

　　5.全力办好民生实事。按照普惠制和择急择重、量力而行、尽力而为的原则，全力办好为沿山贫困村投放小尾寒羊、农户科学储粮仓建设、人畜饮水、孤老病残及农民工等群体法律援助服务、城市公厕建设、农村劳动力技能提升培训、高校毕业生就业扶持、便民市场和夜市建设、农村互助老人幸福院和“乡村舞台”建设等民生实事，让人民群众共享改革发展成果。

　　6.加强和创新社会治理。坚持系统治理、依法治理、综合治理、源头治理，完善基层社会服务管理体系，深化网格化服务管理工作。全面推行社会稳定风险评估机制，创新完善多元化矛盾纠纷排查调处体系，强化人民调解、行政调解、司法调解联动工作体系，建立健全畅通有序的诉求表达、矛盾调处、权益保障机制。深入推进“平安张掖”建设，严密防范、坚决打击敌对势力的渗透破坏活动，依法打击各类违法犯罪活动，维护国家安全和社会大局和谐稳定。全面落实安全生产责任，加大重点行业和重点领域安全隐患排查整治力度，有效防范和坚决遏制重特大安全事故发生。加强产品质量安全管理，建立最严格的覆盖全过程的食品药品监管、原产地可追溯制度，保障人民群众饮食用药安全。全面推进民族团结进步市和“两个共同”示范县建设，依法管理宗教事务。支持工会、共青团、妇联、工商联等群团组织工作，充分激发社会组织活力。

　　全面深化改革和加强政府自身建设

　　以深化改革总揽政府工作全局，切实转变政府职能，着力建设法治政府、责任政府、廉洁政府和服务型政府，增强公信力和执行力。

　　积极稳妥推进各项改革。深入贯彻党的十八届三中全会精神和省委、省政府决策部署，按照市委《关于全面深化改革的安排意见》，坚决把中央和省上已经明确的改革任务落到实处。分类推进当前即改、能改事项的改革，加快落实工商注册便利化、竞争性环节价格市场化和财政预决算、“三公经费”、部门预决算公开等各项改革任务，用改革的思路和办法破解发展难题，充分激发各类市场主体活力。紧密结合张掖实际，研究深化改革的重点内容、方法步骤和具体措施，在政府投入体制、资源整合机制、工程项目代建、社会中介组织管理等重要领域，明确具体的改革目标任务。在农村宅基地使用权抵押、城乡保障政策衔接等领域确定若干试点，大胆探索，稳妥推进，积累经验。按照精简、效能的原则，高质量完成市、县(区)两级政府机构改革任务，确保编制不增长、机构不膨胀、经费不增加、履职更到位。加快推进事业单位分类改革，全市一律停止新增自收自支事业编制，推动有条件的事业单位转为企业或社会组织。

　　推进公共资源配置市场化。全面盘点清理全市公共资源，进一步完善公共资源交易有形市场，尽可能减少政府对资源的直接配置，充分发挥市场在资源配置中的决定性作用。最大限度减少审批和行政事业性收费，严禁政府部门通过中介机构和行业协会变相设立审批收费事项，把该管的事务管住、管好，把该放的事务放开、放活。鼓励民间投资主体参与国有企业改制重组和重大项目建设，推进国有资本和非公有制资本相互融合，促进各种所有制经济共同发展。大胆推进混合所有制经济发展，对由政府出资建设的景区景点和旅游设施、项目，通过公开出售、吸引民间资本参股等多种形式，化债权债务为股权股份，变资产管理为资本管理，实现国有资产保值增值、经营性项目效益提升。加强市、县(区)融资平台管理，建立完善的现代企业制度和科学严格的绩效考核制度，定期审计监督，防范和化解债务风险。

　　加快转变政府职能。进一步加强政府治理体系和行政能力建设，加快推进政府职能转变。选择重点部门、单位先行试点，对政府职能、权力进行全面清理，对各类审批的重要性进行评估，采取倒逼机制，列出负面清单向社会公示，广泛接受群众监督。运用现代管理技术，扎实推进政务服务标准化、集约化，分层次、分类别科学设计办理流程，缩短审批时限，提高工作效率。把决策咨询、公众参与、合法性审查和集体讨论作为决策必经程序，严格依照法定权限和程序行使权力，确保政府决策规范、科学、高效。全面落实服务承诺制、岗位责任制、限时办结制、首问负责制和失职追究制，提高“一站式”服务水平。把提高执行能力和推动工作落实作为市、县(区)两级政府转变作风的核心任务和关键措施，继续坚持和完善领导干部牵头招商引资、联系重大项目、包抓重点信访案件责任制，形成有职有责、在岗尽责的责任落实机制。完善政府目标责任制检查考核办法，严明奖罚，推动各项工作高效落实，确保任务目标如期实现。

　　加强廉洁勤政政府建设。扎实开展党的群众路线教育实践活动，广泛听取群众意见，着力解决“四风”方面存在的突出问题。严格执行中央、省市委各项廉政规定，切实推进惩治和预防腐败体系建设，健全风险预警和权力运行监控机制，强化对重要部门、重要岗位和关键环节权力运行的制约监督。抓紧建立全面规范、公开透明的现代财政制度，从紧从严控制一般性支出。扩大部门预算公开范围，进一步增强预算编制的完整性、准确性、公开性和执行的严肃性。认真贯彻落实《党政机关厉行节约反对浪费条例》，把有限的资金用在推动发展、改善民生、增进群众福祉上。加大对公共资金使用、公共权力运行和公共部门履职尽责情况的监督检查力度，严肃查处各类违纪违法案件，依法纠正和治理损害群众利益的不正之风。深入推进机关“效能风暴”行动，围绕政府机构改革、职能转变和生态安全、安全生产、食品安全及扶贫资金的管理使用，开展效能监察、执法监察和廉政监察，强化问责追究，严肃查处行政不作为、乱作为、慢作为行为，形成风清气正劲足的良好局面。

　　各位代表，新形势、新任务对我们提出了新的更高要求。让我们在省委、省政府和市委的坚强领导下，同心同德、真抓实干，锐意创新、开拓进取，为“建设丝路明珠金张掖、实现幸福美好家园梦”而努力奋斗!
